# Scalable, cGMP-compatible purification of extracellular vesicles carrying bioactive human heterodimeric IL-15/lactadherin complexes

**DOI:** 10.1080/20013078.2018.1442088

**Published:** 2018-02-28

**Authors:** Dionysios C. Watson, Bryant C. Yung, Cristina Bergamaschi, Bhabadeb Chowdhury, Jenifer Bear, Dimitris Stellas, Aizea Morales-Kastresana, Jennifer C. Jones, Barbara K. Felber, Xiaoyuan Chen, George N. Pavlakis

**Affiliations:** ^a^ Human Retrovirus Section, Vaccine Branch, Center for Cancer Research, National Cancer Institute at Frederick, Frederick, MD, USA; ^b^ Laboratory of Molecular Imaging and Nanomedicine, National Institute of Biomedical Imaging and Bioengineering, National Institutes of Health, Bethesda, MD, USA; ^c^ Human Retrovirus Pathogenesis Section, Vaccine Branch, Center for Cancer Research, National Cancer Institute at Frederick, Frederick, MD, USA; ^d^ Vaccine Branch, Center for Cancer Research, National Cancer Institute, Bethesda, MD, USA

**Keywords:** Exosomes, clinical grade, immunotherapy, bioreactor, purification, large scale, tangential flow filtration, size-exclusion chromatography, interleukin-15, lactadherin, Kenneth W. Witwer, Johns Hopkins University, USA

## Abstract

The development of extracellular vesicles (EV) for therapeutic applications is contingent upon the establishment of reproducible, scalable, and high-throughput methods for the production and purification of clinical grade EV. Methods including ultracentrifugation (U/C), ultrafiltration, immunoprecipitation, and size-exclusion chromatography (SEC) have been employed to isolate EV, each facing limitations such as efficiency, particle purity, lengthy processing time, and/or sample volume. We developed a cGMP-compatible method for the scalable production, concentration, and isolation of EV through a strategy involving bioreactor culture, tangential flow filtration (TFF), and preparative SEC. We applied this purification method for the isolation of engineered EV carrying multiple complexes of a novel human immunostimulatory cytokine-fusion protein, heterodimeric IL-15 (hetIL-15)/lactadherin. HEK293 cells stably expressing the fusion cytokine were cultured in a hollow-fibre bioreactor. Conditioned medium was collected and EV were isolated comparing three procedures: U/C, SEC, or TFF + SEC. SEC demonstrated comparable particle recovery, size distribution, and hetIL-15 density as U/C purification. Relative to U/C, SEC preparations achieved a 100-fold reduction in ferritin concentration, a major protein-complex contaminant. Comparative proteomics suggested that SEC additionally decreased the abundance of cytoplasmic proteins not associated with EV. Combination of TFF and SEC allowed for bulk processing of large starting volumes, and resulted in bioactive EV, without significant loss in particle yield or changes in size, morphology, and hetIL-15/lactadherin density. Taken together, the combination of bioreactor culture with TFF + SEC comprises a scalable, efficient method for the production of highly purified, bioactive EV carrying hetIL-15/lactadherin, which may be useful in targeted cancer immunotherapy approaches.

## Introduction

Small extracellular vesicles (EV) comprise a diverse class of nanosized particles (30–200 nm) produced by cells throughout the body, and include exosomes and microvesicles [1]. Not only do EV play an important role in facilitating intercellular communication in homeostasis, but they also affect immunological pathways [2], cancer progression [3] and treatment response [4]. The functional characteristics of EV lend themselves to diverse clinical applications including diagnostics, prognostics and therapeutics. Immunomodulation using natural or engineered EV, especially for the treatment of cancer, is being explored by multiple groups [5]. Notably, several clinical trials have tested primary human dendritic cell-derived EV as a cell-free cancer vaccine platform [6,7]. One of the immunostimulatory effects associated with dendritic cell EV was direct activation of NK cells through NKG2D and interleukin-15 (IL-15) pathways [8].

IL-15 is an immunostimulatory cytokine that is co-expressed with a stabilizing transmembrane polypeptide, IL-15 receptor alpha (IL-15Rα), forming a tight complex (heterodimeric IL-15; hetIL-15) within the endoplasmic reticulum of the secreting cell [9]. This complex is transported to the plasma membrane, where it can bind and signal to responding cells (primarily cytotoxic lymphocytes) expressing the βγ IL-2/IL-15 common receptor [10]. The IL-15:IL-15Rα membrane bound complex is then cleaved, resulting in bioactive, secreted hetIL-15, which can be detected in blood [11]. hetIL-15 has pleiotropic effects on NK and T cells, including the induction of proliferation and phenotypic maturation [12,13], and is associated with immune-mediated antitumor effects in mouse models of cancer [14].

In our previous work, we found that hetIL-15 is also present on the surface of EV, which could potentially be used as delivery vehicles to enhance accumulation of the cytokine within tumors [15]. In that study, we cultured recombinant HEK293 cells expressing hetIL-15 in a hollow-fibre bioreactor with serum-free media, purified EV by ultracentrifugation (U/C), and found that yield of EV was consistently >40-fold higher than conventional tissue culture flasks.

In the current study, we focused on tackling two additional challenges in the translational development of therapeutic hetIL-15 EV, namely the need for increased loading of EV with cytokine, and that of a more scalable and robust purification strategy for bioreactor-conditioned media.

While we initially relied on natural trafficking of hetIL-15 to the vesicular membrane, we looked for ways to improve EV-targeted expression. We focused on generating gene constructs fused to EV-enriched proteins, as a way to bioproduce EV carrying complex macromolecules. Engineered EV have utilized Lamp2b, CD63, or other EV-targeted protein constructs to preferentially express peptides (e.g. RVG) or proteins (e.g. GFP) on the EV surface [16,17]. Fusion to mouse lactadherin has been an especially promising method of loading target immunoregulatory molecules on the EV surface [18]. Lactadherin has been shown to bind to phosphatidylserine-containing vesicles via its C1C2 domain [19]. As an example, a streptavidin/lactadherin construct was expressed on B16BL6 cell-derived EV and was able to stably bind an ^125^I-labelled biotin derivative for effective quantitative assessment of EV biodistribution [20].

For purification of clinical-grade EV, previous reports employed combinations of filtration, polyethylene glycol precipitation and/or U/C [21–23]. Each of these methods presents limitations regarding preparation purity, consistency, efficiency and/or scalability, which may not facilitate large-scale therapeutic EV production and purification. Additional methods are being developed for EV purification, that avoid pelleting of large non-EV components, morphological changes and aggregation which have been reported to occur with U/C-based protocols [24]. To achieve greater purity without the need to pellet EV, size-exclusion chromatography (SEC) has been employed [25–27]. To overcome the volume limitation of chromatography columns, centrifugal ultrafiltration was employed in some of these and other studies [28] to concentrate the starting material. Tangential flow filtration (TFF) may also be used as a method to process even larger volumes of conditioned media for EV purification [29]. TFF is a rapid processing method whereby sample flow is directed in parallel to a semipermeable membrane, allowing for buffer exchange by diafiltration as well as concentration of sample with simultaneous removal of non-EV components. Importantly, use of TFF to concentrate-conditioned media prior to SEC may remediate the inherent limitations of column loading volume, which restricts batch volume and increases the processing time and costs associated with purification.

Herein we present an efficient and robust method for the production and purification of EV, using scalable, cGMP-compatible technologies. Cells stably expressing a fully human hetIL-15/lactadherin-fusion protein were established and propagated within a hollow-fibre bioreactor to produce a continuous supply of EV-rich supernatant. We compared SEC with U/C preparations of EV and demonstrated significant differences in protein content between processing methods. We then prepared highly concentrated conditioned media by TFF followed by purification with SEC to validate a scalable method for the production of highly purified, bioactive EV for therapeutic testing.

## Materials and methods

We have submitted all relevant data of our experiments to the EV-TRACK knowledgebase (EV-TRACK ID: EV170014)[30].

### Generation of hetIL-15/lactadherin DNA plasmid

An optimized DNA vector encoding secreted human hetIL-15, a complex consisting of IL-15 and the IL-15Rα ectodomain, was previously generated in our lab [9]. This plasmid was used as the backbone for the novel chimeric hetIL-15/lactadherin construct.

Alignment of the mouse (P21956) and human (Q08431) lactadherin proteins (Uniprot database; www.uniprot.org, accessed 10 April 2015) identified the human regions homologous to the mouse EGF-like domain C-terminus and C1C2 domains (**Figure S1**). Human codon-optimized DNA (GeneArt, ThermoFisher) encoding this region of human lactadherin was inserted at the 3ʹ end of the human IL-15Rα ectodomain. The amino acid sequence of the resulting hetIL-15α/lactadherin-fusion protein (encoded by plasmid no. AG304) is provided in **Figure S2**. Endotoxin-free AG304 DNA was prepared for subsequent experiments (Plasmid Maxi Kit, Qiagen).

### Conventional cell culture

HEK293H cells were obtained from Gibco (ThermoFisher), and used to generate the cell clones for EV production in this study. For conventional culture (tissue culture flasks and petri dishes), HEK293 cells were grown in DMEM, supplemented with 10% fetal calf serum and 100 U/mL penicillin/streptomycin. For EV harvests from conventional cell culture, EV-depleted cell culture medium was used, as previously described [15]. Briefly, EV were depleted from complete cell culture medium by ultrafiltration using a 500 kDa MWCO tangential flow filter device (mPES MidiKros, SpectrumLabs). Cells were seeded in conventional media, and incubated overnight to allow for attachment. Subsequently, medium was removed, the monolayer gently washed with PBS and EV-depleted media added for 48 h, at which time conditioned media was harvested for immediate downstream processing.

NK92 cells were a kind gift of Dr. Howard A. Young (Cancer and Inflammation Program, National Cancer Institute, USA). NK92 cells were cultured in RPMI 1640 supplemented with 10% fetal calf serum,100 U/mL penicillin/streptomycin, 200 U/mL recombinant IL-2 (National Cancer Institute) and 10 ng/mL hetIL-15 (Admune Therapeutics, Danvers, MA).

### Cell line generation

Generation of HEK293 cells stably expressing full-length human hetIL-15 (clone 19.7) was previously reported [9].

For this study, HEK293 cells stably expressing the novel hetIL-15/lactadherin complex were generated. HEK293 cells were transfected with 10 µg linearized AG304 plasmid and 1 µg linearized neomycin resistance plasmid, using calcium phosphate transfection method. Cell culture medium supplemented with 500 µg/mL neomycin (G418; KSE Scientific) was used to select for transfected cells. Individual clones were screened for both total hetIL-15 secretion and EV-associated hetIL-15 by ELISA (human IL-15 Quantikine, R&D Systems). A high-producing cell clone (no. 159) was expanded and seeded in the hollow-fibre bioreactor, as described later. All cell lines used in this study were tested for mycoplasma contamination, and were found to be negative, using the MycoAlert assay (Lonza).

### Hollow-fibre bioreactor cell culture

HEK293 cell clones stably expressing hetIL-15/lactadherin, hetIL-15 or no IL-15 (control) were seeded in separate hollow-fibre bioreactors (C2011; Fibercell Systems), and maintained in serum-free medium as previously described [15]. Briefly, cells were seeded in the extracapillary space (ECS) of a culture cartridge containing densely packed hollow fibres. Feeding medium was constantly circulated through the lumen of the hollow fibres via a pump system. Nutrients/waste could move freely across the hollow-fibre membrane (20 kDa MWCO), while large cellular products (including EV) accumulated in the ECS. 20 mL of ECS was harvested five times weekly, immediately centrifuged at 300 × *g* for 7 min, then 3,000 × *g* for 15 min and stored at −80°C for batch processing.

### EV purification

EV from hollow-fibre, bioreactor-conditioned media were purified either by U/C or SEC. For both methods, clarified-conditioned medium (CCM) was generated by sequential centrifugation (300 × *g* for 7 min; 3,000 × *g* for 15 min; 20,000 × *g* for 45 min), followed by 0.22 μm filtration (Stericup; EMD Millipore) to remove cell debris and large EV. Conventional culture CCM was then centrifuged at 110,000 × *g* for 3 h (SW40 rotor; Beckman Coulter) and the EV pellet was resuspended in PBS. Bioreactor culture CCM processed by U/C was centrifuged at 110,000 × *g* for 2 h (70.1 Ti rotor; Beckman Coulter). The EV pellet was resuspended in TBS to the original sample volume, and centrifuged again to obtain a washed EV pellet (resuspended in PBS) using a syringe with a 27 G needle. Resuspended EV pellet was clarified of any remaining aggregates by a short (3 min) centrifugation at 18,000 × *g*.

For SEC purification, CCM was injected in a pre-packed, preparative-grade Superdex 200 column (GE Healthcare) attached to an HPLC apparatus (Dionex UltiMate 3000; Fisher Scientific). Specifically, 0.5 mL CCM was injected in a pilot-scale 30 cm column (Increase 10/300 GL model, 1 cm diameter, 24 mL bed volume) or 4–5 mL CCM was injected in a larger-scale 120 mL column (HiLoad 16/600 model). Isocratic elution of the column with sterile-filtered PBS took place over a period of 30 and 120 min, at a constant flow rate of 1 and 1.6 mL/min, respectively for the smaller and larger columns. Fractions were collected at 1 min intervals. Column pressure and UV-light absorbance (260, 280 nm) were monitored throughout the elution time. The columns were washed with one column volume of NaOH (500 mM) between runs and further washed with two column volumes of 30% ethanol before storage of the column at 4°C. The column was equilibrated with at least two column volumes of sterile-filtered PBS prior to applying the samples.

### TFF

TFF was used in indicated experiments to remove non-EV components from CCM and for subsequent concentration.

In small-scale experiments comparing different MWCO filters, media was gently circulated through 750 kDa and 0.05 μm microKros devices (SpectrumLabs) by manual pumping with 20 mL syringes affixed to either end of the filter devices. Initially, 6 mL CCM was diluted with an equal volume of PBS, and pumped back and forth across the filter device until the volume decreased to 6 mL (due to ultrafiltration of components smaller than the MWCO). Again, 6 mL PBS was added to re-dilute the conditioned medium, which was then manually pumped through the filter device. This process of isovolumetric ultrafiltration was repeated five times, at which point 1 mL was sampled from the 6 mL processed conditioned medium (1× [unconcentrated], TFF-processed sample). The remaining 5 mL were subsequently concentrated to approximately 1–1.5 mL by additional pumping through the filter device. This resulted in a concentrated (~5×), TFF-processed sample. A matching pre-TFF sample was also generated, and subjected to downstream analysis as an experimental control for the TFF-processed samples.

For the larger-scale EV purification experiments (>200 mL), a polysulfone (PS) MidiKros TFF device (0.05 μm pore size, SpectrumLabs) was incorporated into a tubing circuit, which was connected to two inlets of a triple-inlet reservoir, containing CCM (**Figure S3**). A PBS reservoir (dialysate) was connected to the sample reservoir via the third inlet. 220 mL CCM were circulated through the TFF device by a peristaltic pump. Since components smaller than the pore size were removed by ultrafiltration, negative pressure developed within the media reservoir, resulting in an equal volume of PBS being drawn in from the dialysate reservoir. This process of isovolumetric ultrafiltration was continued for a total of four-volume buffer exchanges with PBS, as a means of removing significant amounts of non-EV contaminants. Next, the PBS reservoir was detached and the TFF-processed conditioned medium was additionally circulated through the TFF device until the indicated level of concentration was achieved. Throughout the process, circulation rate was adjusted to maintain pressure in the TFF device to <20 psi (as measured by an in-line analog pressure monitor).

### EV composition characterization

Protein concentration of samples was determined by Bradford assay. In experiments where one or more samples had a protein concentration below the limit of detection (i.e. <125 μg/mL), samples were concentrated by ultrafiltration using an Amicon-Ultra microconcentrator (EMD Millipore; 3 kDa MWCO; centrifugation of 500 μL sample for 20 min, at 14,000 × *g*). 5 μL of each sample or protein standard dilution (bovine γ-globulin) was added to 250 μL of Quickstart Bradford Reagent (Biorad) in wells of a 96-well plate. Protein concentration was estimated based on O.D. at 595 nm using a microplate reader (SpectraMax M3; Molecular Devices).

Commercial ELISA kits were used to determine the concentration of human ferritin (cat. no. ab200018, Abcam) and human IL-15 (cat. no. D1500, R&D Systems).

For mass spectrometry, 20–30 μg of EV were lysed by addition of 5× RIPA buffer and run on SDS-PAGE. The gel was stained with SimplyBlue Safestain (Invitrogen), and individual lanes were digested overnight with trypsin (Promega) at 37ºC. Samples were desalted by C18 ZipTip (Millipore), lyophilized and resuspended in 0.1% formic acid for analysis by LC-MS. Each sample was loaded on an Easy nLC 1200 nano-capillary HPLC system (Thermo Scientific) with a C18 Nano Trap Column, (Acclaim PepMap100 C18, 2 cm, nanoViper, Thermo Scientific) and an analytical column (Acclaim PepMap RSLC C18, 15 cm, nanoViper, Thermo Scientific) connected with a stainless-steel emitter, coupled online with a Q Exactive HF hybrid OrbiTrap mass spectrometer (Thermo Scientific) for analysis by RPLC-MS/MS. A linear gradient of 2% mobile phase B (acetonitrile with 0.1% formic acid) to 42% mobile phase B was used to elute peptide over a period of 70 min, using a flow rate of 200 nL/min. The 12 most intense molecular ions in the MS scan were selected for high-energy collisional dissociation (HCD) using a normalized collision energy of 30%. The mass spectra were acquired at the mass range of m/z 300–2000. Capillary voltage and temperature were set at 1.7 kV and 275°C, respectively (Easy Nano Spray ion source; Thermo Scientific). Dynamic exclusion was enabled on the mass spectrometer during the MS2 data acquisition. Spectra were searched against the human UniProt database downloaded from the European Bioinformatics Institute website (http://www.ebi.ac.uk/integr8), January 2017, utilizing Proteome Discoverer 1.4 (Thermo Scientific). Up to two missed tryptic cleavage sites were allowed during the database search, while the oxidation (+15.9949 Da) of methionyl residues was included as a possible dynamic modification. The data was searched with a precursor ion tolerance of 20 ppm and a fragment ion tolerance of 50 ppm. The peptide identifications were filtered through a protein percolator with the cutoff of a false peptide discover rate (FDR) less than 1% for all peptides identified. “Strict Maximum Parsimony Principle” was applied during the data compiling.

For gene-set enrichment analysis (GSEA), mapped proteins were divided into three groups: proteins unique to either SEC or U/C EV, and shared proteins. Each set was analyzed using the online PANTHER overrepresentation test, based on the GO cellular component gene ontology, with Bonferonni correction applied (http://amigo.geneontology.org/amigo, accessed May 2017).

For protein electrophoresis gels, samples were lysed with RIPA buffer for 45 min on ice (Boston Bioproducts). SDS-PAGE gels were run under denaturing, reducing conditions by loading equal volume of samples, unless otherwise specified. Coomassie staining of protein gels was performed with SimplyBlue SafeStain (Invitrogen), according manufacturer instructions. Briefly, gel was incubated in dye for 1 h, washed in dH_2_O for 1 h and washed in 20% NaCl overnight prior to imaging. For Western blots, gels were transferred to nitrocellulose membranes, blocked and probed overnight with anti-CD63 (1:1,000; clone EPR5702, Abcam) or anti-Calnexin (1:20,000; clone EPR3632, Abcam).

For EV flow cytometric analysis, a bead-based commercial kit was used (ExoFlow, System Biosciences Inc.). 20 μg EV were incubated overnight at 4°C with 9 μm beads coated with anti-CD63 antibody included with the kit. Beads were then stained with FITC-conjugated anti-human IL-15 antibody (10 μL per 100 μL reaction; clone 34,559, R&D Systems) and analyzed by flow cytometry.

### EV biophysical characterization and immune-transmission electron microscopy (immune-TEM)

Nanoparticle tracking analysis was used to determine the particle size and concentration of EV preparations. Samples were diluted in PBS, and 5 videos of 30 sec each were acquired on a Nanosight LM10 device (NTA version 3.1, Build 3.1.54; Malvern Instruments). Brightness was set to 14 and detection threshold was set to 6, while blur size and maximum jump distance was set to auto.

EV were prepared for immune-TEM according to the protocol described previously, with minor modification [31]. Briefly, a 10 μL suspension of diluted EV was coated on a Formvar/Carbon 400 mesh copper grid (Ted Pella, Inc.) for 1 h. The grid was washed in PBS three times followed by fixation in 4% paraformaldehyde (EM-grade, Sigma-Aldrich) for 10 min. Grids were washed five times with PBS prior to coating with 10 μL human IL-15 primary antibody (10 μg/mL, MAB247-SP, R&D Systems) for 40 min. Free antibody was then removed and the grid was blocked with three washes of PBS containing 0.1% BSA. Secondary goat anti-mouse IgG gold-labelled (10 nm) antibody (10 μL, ab39619, Abcam) was added to the grid for 40 min followed by three washes with PBS. 2.5% glutaraldehyde (10 μL, EM-grade, Sigma-Aldrich) was used to post-fix EVs for 10 min and was followed by three washes of PBS. EVs were then negatively stained with 2% uranyl acetate (10 μL) for 15 min and further embedded using a 10 μL 0.5% uranyl acetate and 0.13% methyl cellulose solution for 10 min. Sample grids were allowed to dry overnight at room temperature before imaging on a JEOL 2010 TEM operating at an acceleration voltage of 120 keV.

### IL-15 bioactivity assay

IL-15 bioactivity was measured using an *in vitro* bioassay of NK92 cell line proliferation, as previously described [15]. Briefly, EV samples were first assayed by ELISA to determine IL-15 concentration. Next, NK92 cells were cultured in the presence of increasing concentrations of IL-15 (0.05–8 ng/mL) in the form of a purified protein standard (hetIL-15 protein, Admune Therapeutics) or associated with an EV sample. EV lacking IL-15 were used as a negative control. After 72 h, MTT reagent assay (Roche) was used to assess the dose-dependent proliferation of NK92 cells in response to IL-15. The bioassay was performed in triplicate, on two independent assay plates for each sample type.

## Results

### Fusion of hetIL-15 with human lactadherin significantly increases EV-associated cytokine

**Figure 1.**
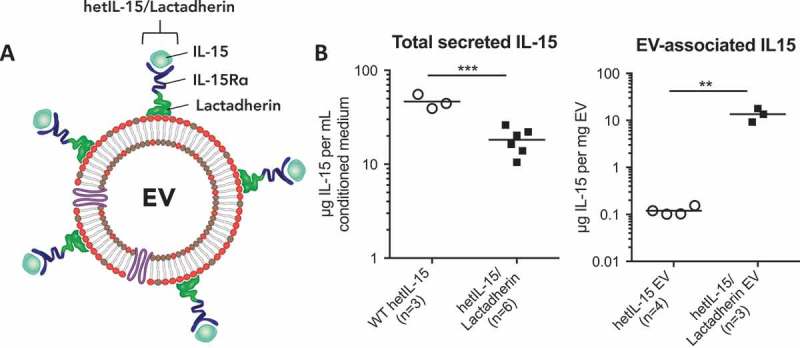
**hetIL-15/lactadherin-fusion protein increases cytokine association with EV**. HEK293 cells were stably transfected with expression vectors for wildtype (WT) heterodimeric interleukin-15 (hetIL-15) or a hetIL-15/lactadherin-fusion construct. Cells were grown in a hollow-fibre bioreactor, and EV were purified from conditioned medium of the bioreactor by differential ultracentrifugation (U/C). (a) Schematic representation of hetIL-15/lactadherin protein complexes bound to the lipid bilayer of an EV via non-covalent interaction with phosphatidylserine. (b) Quantification of total IL-15 secreted in conditioned medium (left panel) and EV-associated IL-15 (right panel) obtained from independent bioreactor harvests; statistical analysis was by *t*-test. ** and *** denote *p *< 0.01 and 0.001, respectively. Horizontal line at group mean.

We previously reported the presence of hetIL-15 on EV purified from stably transfected HEK293 cells expressing the membrane-embedded human cytokine [15]; however, the relatively low levels of EV-associated cytokine suggested a need for improved loading of hetIL-15. Fusion of target proteins to mouse lactadherin has been shown to increase EV association, due to the binding of this protein to phosphatidylserine, a lipid that is enriched in EV [18]. To maximize the amount of hetIL-15 associated with EV, we generated a DNA construct encoding a fully human, chimeric molecule consisting of the IL-15Rα ectodomain fused at its C-terminus with the C1C2 domains of lactadherin. By amino acid sequence alignment, the human homologous C1C2 domain was identified as spanning residues 70–387 (**Figure S1**), matching the domain designation on Uniprot. We additionally included the 19 amino acids preceding the identified C1C2 domains in our IL-15Rα/lactadherin-fusion construct (**Figure S2**). This novel fusion construct was incorporated into a dual-promoter plasmid that co-expressed optimized human IL-15 from the simian CMV promoter (**Figure S2**), resulting in the production of the human hetIL-15/lactadherin complex (Figure 1(a)). To address whether this form of IL-15 would increase the amount of EV-associated cytokine, we generated individual HEK293 cell clones stably expressing high levels of hetIL-15/lactadherin. Clone 159 was expanded in a hollow-fibre bioreactor in serum-free medium, and IL-15 production was compared to that found from HEK293 clone 19.7 expressing the wildtype membrane-embedded human cytokine. The total amount of secreted IL-15 was approximately twofold higher in HEK293 cells expressing the wildtype cytokine (Figure 1(b), **left panel**). However, the amount of IL-15 associated with EV from cells expressing hetIL-15/lactadherin was ~100-fold greater (Figure 1(b), **right panel)**, indicating that fusion with human lactadherin C1C2 domains increased EV loading.

### SEC yields higher quality EV preparations than U/C, without sacrificing yield

In our previous report of efficient EV production and purification, we used U/C to generate EV preparations [15]. However, this method has limited scalability due to long centrifugation times and volume specifications of available commercial rotors. In addition, achieving superior purity (such as by removal of large non-EV protein complexes) requires more complex processing, for example by sucrose gradient U/C [21].

We tested SEC using a commercially available, pre-packed Superdex column as an alternative method of EV purification. To pilot this method, bioreactor-conditioned medium was clarified by low-speed centrifugation and filtration, and then processed through a 10mm × 300 mm preparative-grade column (0.5 mL per chromatography run). By monitoring the UV absorbance of the SEC eluate, we observed an early A260/A280 peak, along with a series of peaks at later time points (see **Figure S4**). We anticipated that EV would be found primarily in the early peak, given the large size of EV relative to other macromolecular components secreted by cells. Indeed, the majority of EV eluted in a single fraction (F9) using this setup. For subsequent experiments with the pilot-scale SEC column, F9 was considered as the purified EV preparation, unless otherwise indicated.

**Figure 2.**
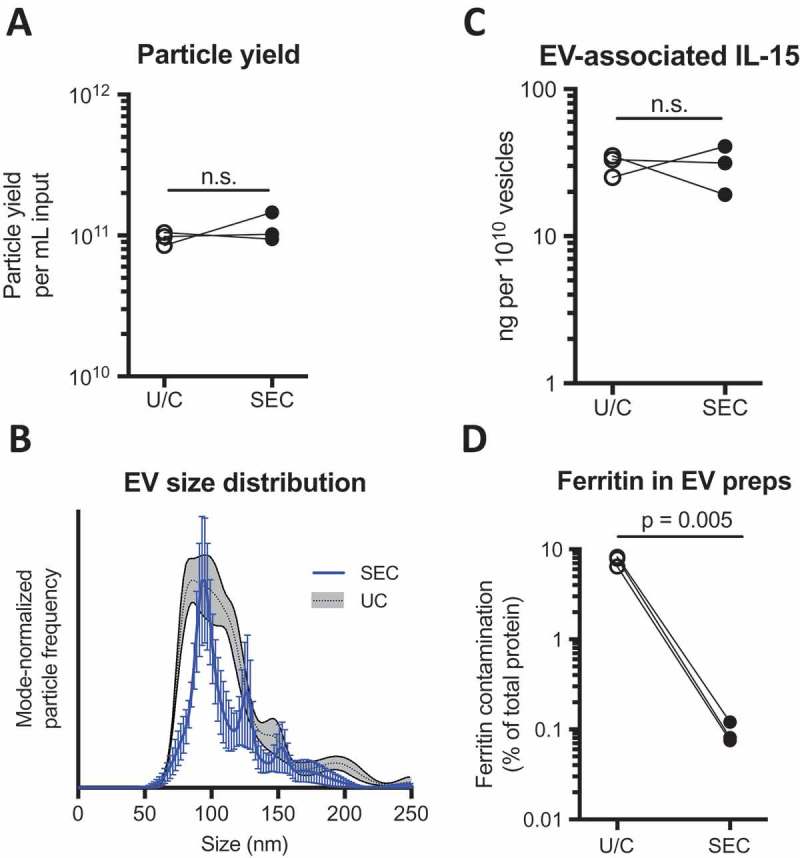
**Size-exclusion chromatography (SEC) efficiently yields hetIL-15/lactadherin EV, and removes large macromolecular protein contaminant**. Three bioreactor harvests were divided and processed independently by either differential ultracentrifugation (U/C) or size-exclusion chromatography (SEC; 30 cm column) for the purification of EV. (a) Particle yield of purification methods, per mL input of conditioned medium, as measured by NTA. (b) Representative size distributions of purified EV, assessed by NTA (see **Figure S5** for additional paired EV preps). (c) EV-associated IL-15 after purification, as measured by ELISA. (d) Previous studies identified ferritin as a major macromolecular contaminant of EV preparations. Contamination of EV preparations with ferritin complexes compared between the two purification methods by ELISA. Statistical analyses were by paired *t*-test; n.s. denotes non-significant *p*-value.

We next selected three independent harvests of bioreactor-conditioned medium from cultures of cells expressing hetIL-15/lactadherin, and divided each harvest; one part of each harvest was processed by SEC (0.5 mL per chromatography run), while the other aliquot was processed by U/C for comparison (5 mL per U/C sample). We found that SEC purification produced the same amount of EV per mL input medium as did pelleting by U/C, and these vesicles had a similar size distribution by NTA (Figure 2(a,b) **and S5**). In addition, the amount of hetIL-15 detected in association with EV preparations was also comparable between purification methods (Figure 2(c)).

We also performed comparative proteomics by LC-MS of EV purified by either method. Analysis revealed that the heavy and light chains of ferritin were the two proteins decreased by the greatest amount by SEC purification versus U/C (**Supplementary Data 1 and Figure S6**). Ferritin chains form a 24-unit protein complex, measuring approx. 8 nm in diameter, which carries iron in biological systems [32]. Given the absence of components >3 kDa in cell culture media used in the bioreactor, ferritin complexes were identified as a by-product of our cell culture conditions. We analyzed our paired EV preparations for contamination by ferritin protein complexes using a commercial ELISA kit, and found that SEC decreased the abundance of this protein by 100-fold as compared to U/C (Figure 2(d)). Analyzing early fractions of SEC eluate, we confirmed that the Superdex column could resolve EV and ferritin complexes, the latter of which eluted in later fractions (**Figure S4**).

Further analysis of our proteomics data by GSEA based on the GO cellular component ontologies [33] revealed that there was a highly significant enrichment of EV/exosome-associated ontologies with both purification methods (**Figure S6**). However, U/C preparations had a large number of unique mapped proteins, which showed statistically significant enrichment association with non-EV ontologies by GSEA.

Considered together with the observed removal of large protein complexes (ferritin) from EV preparations by SEC, these data suggest that U/C preparations were of lower purity. Thus, SEC was able to achieve higher EV purity, without sacrificing EV yield as compared to U/C.

### SEC purification retains hetIL-15/lactadherin complex on the EV surface

HEK293 clone 159 cultured in the hollow-fibre bioreactor stably expressed hetIL-15/lactadherin, a fusion protein that showed increased association with EV preparations. While we showed earlier that SEC does not appear to decrease the association of hetIL-15/lactadherin with EV (as compared to U/C purification), we conducted additional analyses to confirm the surface display of the cytokine on EV.

**Figure 3.**
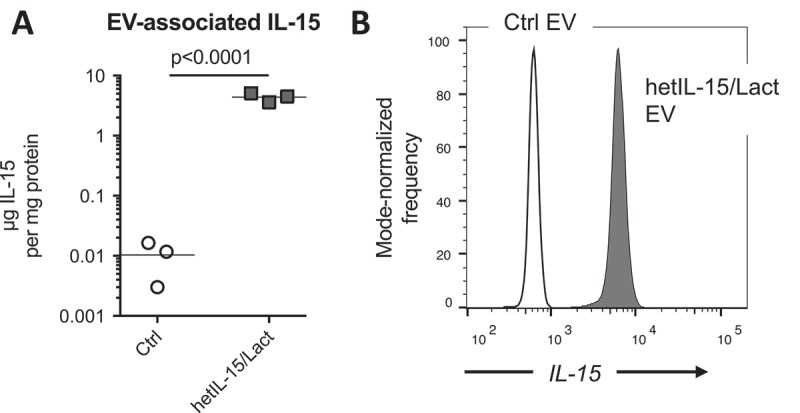
**hetIL-15/lactadherin is retained on the surface of EV purified by SEC**. EV were purified by SEC from three harvests of bioreactor-conditioned medium of control or hetIL-15/lactadherin expressing HEK293 cells. (a) IL-15 levels were measured in EV preps by ELISA. Statistical analysis was by *t*-test; horizontal line at group mean. (b) Surface expression of hetIL-15/lactadherin was confirmed by immobilizing purified EV on anti-CD63 antibody coated beads, and probing with a FITC-anti-IL-15 antibody. EV-coated beads were analyzed by flow cytometry.

By ELISA, we determined that the mean EV loading (after SEC purification) was 4.4 μg IL-15 per mg total EV protein (Figure 3(a)). IL-15 levels associated with control EV purified using the same method were negligible. To confirm the presence of hetIL-15/lactadherin on the surface of EV, we immobilized the vesicles on 9 μm beads coated with anti-CD63 antibody, and probed the intact EV surface with a fluorophore-conjugated anti-IL-15 antibody. Analysis of the beads by flow cytometry detected a strong fluorescent signal associated with the hetIL-15/lactadherin EV, as compared to similarly prepared control EV lacking cytokine expression (Figure 3(b)).

### TFF removes the majority of non-EV components

Having found that SEC is an efficient way to obtain high-purity EV with surface bound hetIL-15/lactadherin, we sought to incorporate a purification step that would allow batch processing and concentration of large volumes of conditioned media. TFF is an ultrafiltration-based procedure that can process volumes from a few mL to a few thousand litres, allowing for rapid buffer exchanges and medium concentration [34]. To assess the effect of TFF on EV yield, we performed pilot experiments using micro-sized TFF devices. Given the large size of EV compared to other conditioned medium components, we tested devices with large pores, either 750 kDa (MWCO) or 50 nm in size. We divided bioreactor-conditioned medium, and processed by SEC alone or TFF + SEC. For TFF-processed samples, we performed isovolumetric buffer exchanges with PBS (generating a 1×, unconcentrated sample) and then proceeded to concentrate by about fivefold (generating a ~ 5× sample).

**Figure 4.**
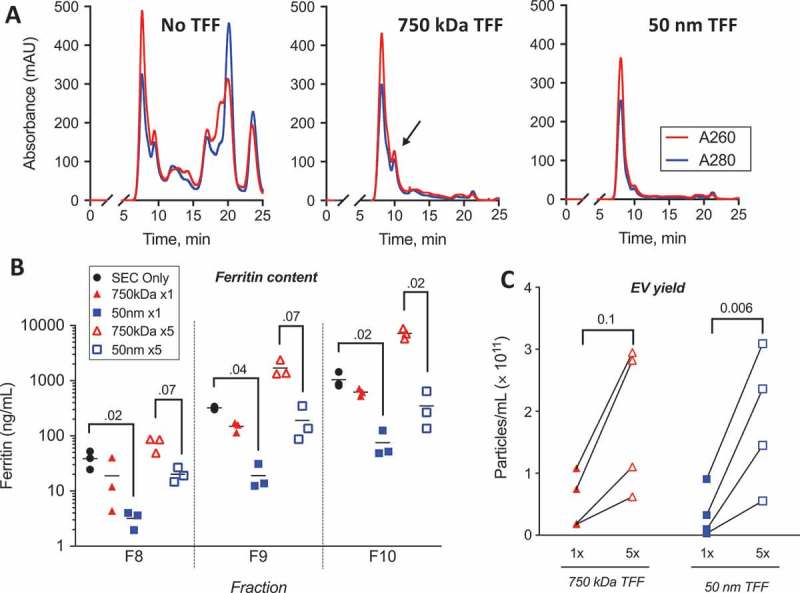
**Tangential flow filtration (TFF) decreases non-EV protein and concentrates EV**. EV were purified from conditioned medium by SEC (30 cm column) with or without prior TFF, with 0.5 mL of each sample injected into the chromatography column. For TFF-processed samples, 6 mL of bioreactor-conditioned medium was manually pumped through the TFF device with the indicated pore size. To achieve isovolumetric ultrafiltration (“1×” samples), medium was repeatedly diluted with PBS and pumped through the device until the original volume was reattained, for a total of five volumes of buffer exchange. Concentrated TFF samples (“5×”) were obtained by additional, continued ultrafiltration to the desired final volume. (a) UV absorbance of chromatography eluate was monitored at 260 and 280 nm wavelengths for SEC alone (left panel), 750 kDa TFF+SEC (middle panel), or 50 nm TFF+SEC (right panel) samples. We confirmed that the first absorbance peak (F8 in these experiments) corresponds to the most EV-rich fraction (**Figure S7**). Black arrow points to 10 min peak that is diminished after 50 nm TFF, likely related to removal of large protein complexes. (b) Removal of large ferritin complexes by 50 nm TFF was quantified in early SEC fractions by ELISA (3 experiments). Friedman test was used to compare measurements within each fraction (independent tests for 1× and 5× samples); p-values <0.1 are shown. (c) TFF concentration of EV was assessed by quantifying EV in F8 from samples subjected to either isovolumetric buffer exchange (unconcentrated, 1× samples) and after approx. 5× concentration.

Running 0.5 mL of each of these samples through the 30 cm SEC column revealed that the first UV-absorbance peak was retained following TFF. As described before, this peak corresponded to eluted EV. However, subsequent peaks were mostly absent following TFF, suggesting significant removal of non-EV components (Figure 4(a)). In addition, TFF with the 50 nm pore-size device appeared to reduce the size of a small peak at 10 min (Figure 4(a), **middle and right panels**). This peak likely corresponded to large protein complexes retained by 750 kDa TFF, but filtered through the 50 nm pores. Knowing that large ferritin complexes were present in the initial bioreactor-conditioned medium, we hypothesized that these complexes would be removed by the 50 nm TFF device, as suggested by the chromatography data. Indeed, analysis of SEC fractions by ELISA revealed that TFF of conditioned medium led to a drop in the concentration of ferritin only by the 50 nm pore device (Figure 4(b)). This was true in both the most EV-rich fraction (F8), as well as in the subsequent fractions that contained higher amounts of this protein complex (F9-F10). By quantifying the EV in F8 using NTA, we found that TFF of either pore size was able to consistently concentrate EV, albeit to a lesser extent than estimated based on the volume concentration achieved (Figure 4(c)). Given the improved results of TFF in larger-scale experiments (see next), we hypothesize this was due to the significant volume of dead-space within the microfilter device in relationship to the total volume of conditioned medium processed. Nonetheless, these preliminary results suggested that TFF was able to concentrate EV and that the 50 nm pore size was more efficient at maintaining a low concentration of large protein complexes, such as ferritin, during the process of concentration. We thus proceeded to scale-up of our purification procedure employing 50 nm pore TFF in subsequent experiments.

### Scale-up of EV production and purification

Having piloted both TFF-concentration and SEC purification of EV at relatively small scales (0.5–2.5 mL batches of medium), we proceeded to demonstrate the scalability of these methods. In these experiments, we used a simple pump-driven TFF apparatus and commercially available midi-scale TFF filters (50 nm pore size) to process higher volumes of bioreactor-conditioned medium (see **Figure S3** for apparatus configuration). For SEC, a larger preparative column (60 cm length, 1.6 cm diameter, 120 mL bed volume) was used.

We started with 220 mL bioreactor-conditioned medium, and performed isovolumetric TFF for a total of four volumes of buffer exchange with PBS, as described in the section “Materials and Methods: TFF” In our pilot experiments (mentioned earlier), we found that this process of isovolumetric ultrafiltration was effective in decreasing non-EV components prior to concentration. Subsequently, the medium was concentrated 16-fold by continuous pumping through the filter device. 4–5 mL of concentrated medium was then processed through the SEC column (equivalent to 64–80 mL of starting material). As a control, 5 mL of bioreactor-conditioned medium was directly run on the SEC column without TFF.

**Figure 5.**
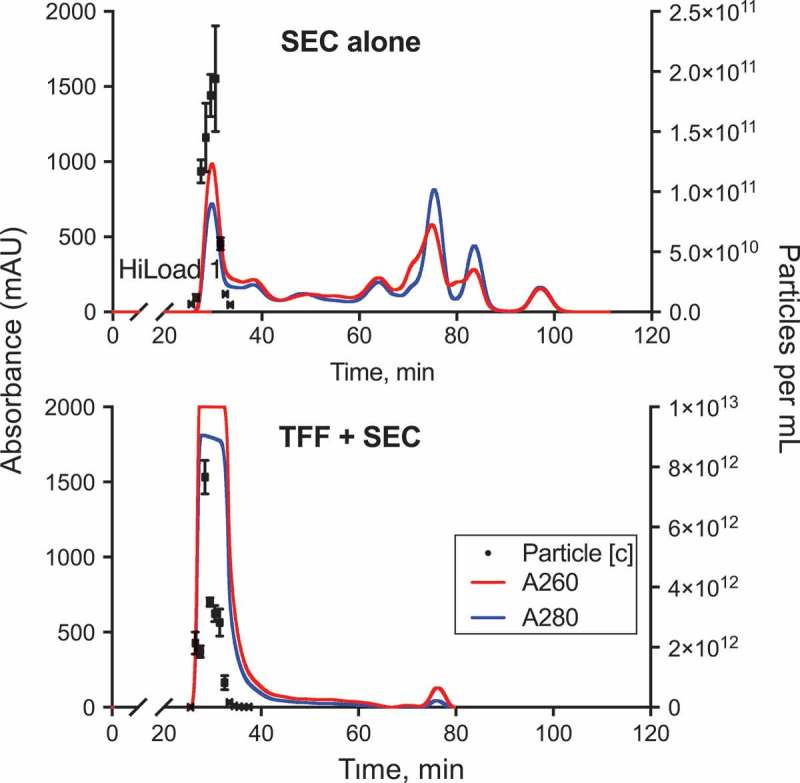
**TFF processing enables scalable EV purification using a large SEC column**. A large 60 cm SEC column was used to purify EV from 5 mL (max sample loading volume) of bioreactor-conditioned medium (top panel). Alternatively, an equal volume of bioreactor-conditioned medium was loaded into the same column after being concentrated by 16-fold using TFF (50 nm pore size) prior to chromatography (bottom panel). See also **Figure S3** for TFF apparatus setup. EV concentration and UV absorbance were monitored in eluted chromatography fractions. Note that the first chromatography peak (EV fractions) of the TFF-concentrated sample led to saturation of the UV-absorbance detector. Subsequent UV-absorbance peaks were diminished in TFF-processed medium.

**Figure 6.**
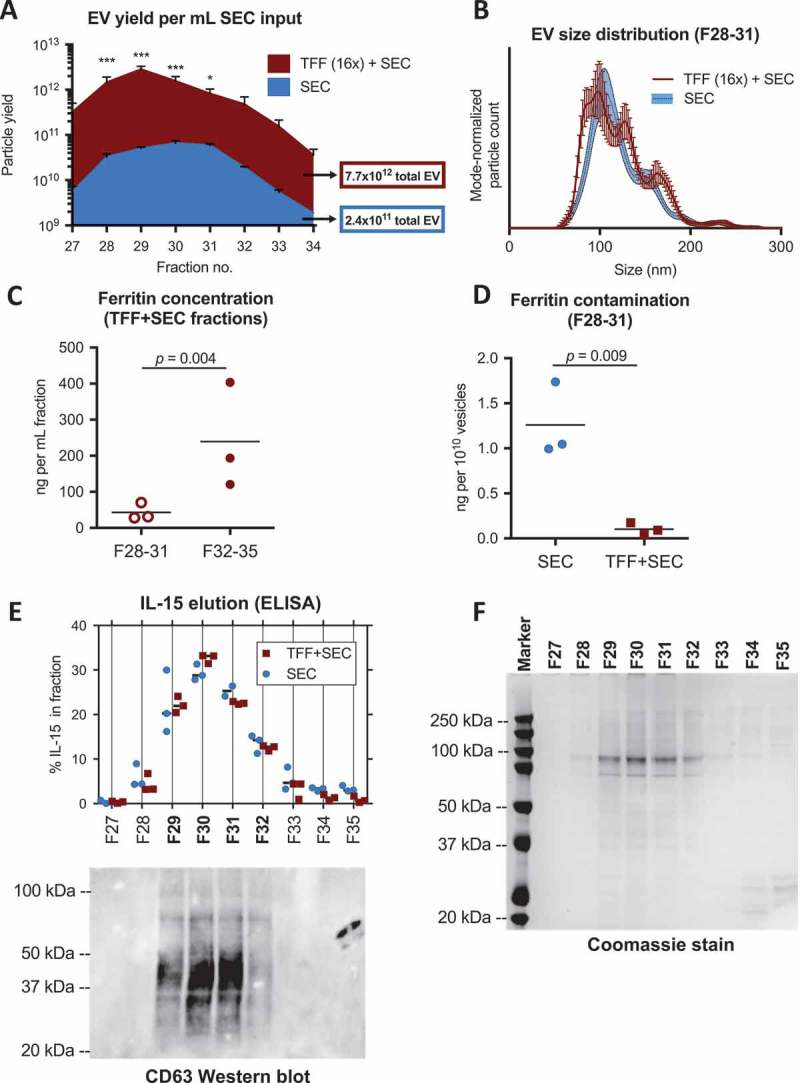
**Large-scale TFF+SEC efficiently yields high-purity EV**. hetIL-15/lactadherin EV were purified from bioreactor-conditioned medium by SEC using a large 60 cm column (3 independent runs). Where indicated, samples were concentrated by 16-fold using TFF prior to SEC purification, as shown in Figure 5. (a) Particle yield in SEC fractions was compared between the two purification methods by NTA (see also **Figure S8**). Total particle yield (per mL sample input) of each method is listed in respectively colored boxes (mean of 3 runs). Statistical analysis was by two-way ANOVA; * and *** indicate *p *< 0.05 and 0.001, respectively. (b) Size distribution of purified EV was found to be similar by NTA, as depicted in representative plots. (c) Ferritin content of the most EV-rich fractions (F28-31) was lower than that of subsequent four fractions (*t*-test). (d) Level of ferritin contamination was lower in EV-rich fractions resulting from TFF+ SEC as compared to SEC alone (*t*-test). (e) IL-15 elution in each fraction was quantified by ELISA, displayed in the upper graph of Panel E as percent of total amount of IL-15 in F27-F35 of each chromatography experimental run. The majority of IL-15 eluted in F29-F32, which correlated with the elution of CD63 (EV-associated protein), as shown in the respective Western blot at the bottom of this panel. (f) Coomassie staining of protein gel from the same fractions (F29-F32) displayed a distinct banding pattern compared to subsequent fractions.

As was the case with the smaller SEC column, we found that EV eluted in early chromatography fractions (Figure 5, **top panel**). While in the smaller SEC column EV eluted mainly in one fraction, we detected EV in a number of fractions eluting from the 60-cm column, perhaps a result of the increased capacity and resolving ability of the larger column. TFF with the 50 nm device led to a significant decrease in subsequent non-EV peaks (Figure 5, **bottom panel**). The first SEC peak (EV-rich fractions) of the TFF-concentrated medium saturated the UV-light absorbance detector of our HPLC apparatus, suggesting that a much larger amount of EV were being eluted. We analyzed early-peak fractions from independent purifications of SEC alone or TFF + SEC by NTA to compare EV yield. There was an overall yield increase of approximately 30-fold when running 16× concentrated medium through the SEC column, which was especially prominent in F28-31 (Figure 6(a)). The size distribution of EV purified in F28-31 by either SEC alone or TFF + SEC was very similar (Figure 6(b)). We next quantified the ferritin content of TFF + SEC fractions, as an indicator of macromolecular impurities in EV-rich fractions. Ferritin concentration was five times lower in fractions containing the greatest amount of EV (F28-31), as compared to subsequently eluted fractions (F32-35; Figure 6(c)). This result indicated that large-scale SEC allowed for significant separation between the majority of EV and large protein complexes. Moreover, comparing the relative content of ferritin in F28-31 (normalized to number of EV), it was evident that TFF decreased the contamination of EV preparations with this large protein complex by at least 10-fold (Figure 6(d)). As a further characterization of the most EV-rich fractions of TFF + SEC, we performed Western blots for CD63 (EV-associated marker) and calnexin (cell-associated marker) (**Figure S9**). We were able to detect both low and high molecular weight bands of CD63, reflecting differential glycosylation as previously described [35]. While EV-rich fractions were enriched for CD63 (compared to cell lysate), F28 had lower levels of this protein, perhaps reflecting elution of different EV subtypes (**Figure S9**). Calnexin was absent from any EV fractions, confirming the absence of enrichment of non-EV cellular components by our methods. Given that the purified EV in these studies were produced by cells expressing hetIL-15/lactadherin, we compared the elution profile of IL-15 versus that of CD63 in early chromatography fractions (Figure 6(e)). We found that the vast majority of IL-15 was present in F29-32, which were the fractions also containing the highest amount of CD63. Only minimal IL-15 was detected in fractions negative for CD63. This suggested that the majority of cytokine was co-purifying, and likely associated with CD63^+^ EV. Coomassie staining of the early chromatography fractions identified a distinct banding pattern in F29-32 (Figure 6(f)), further supporting that these fractions were compositionally distinct from adjacent fractions.

**Figure 7.**
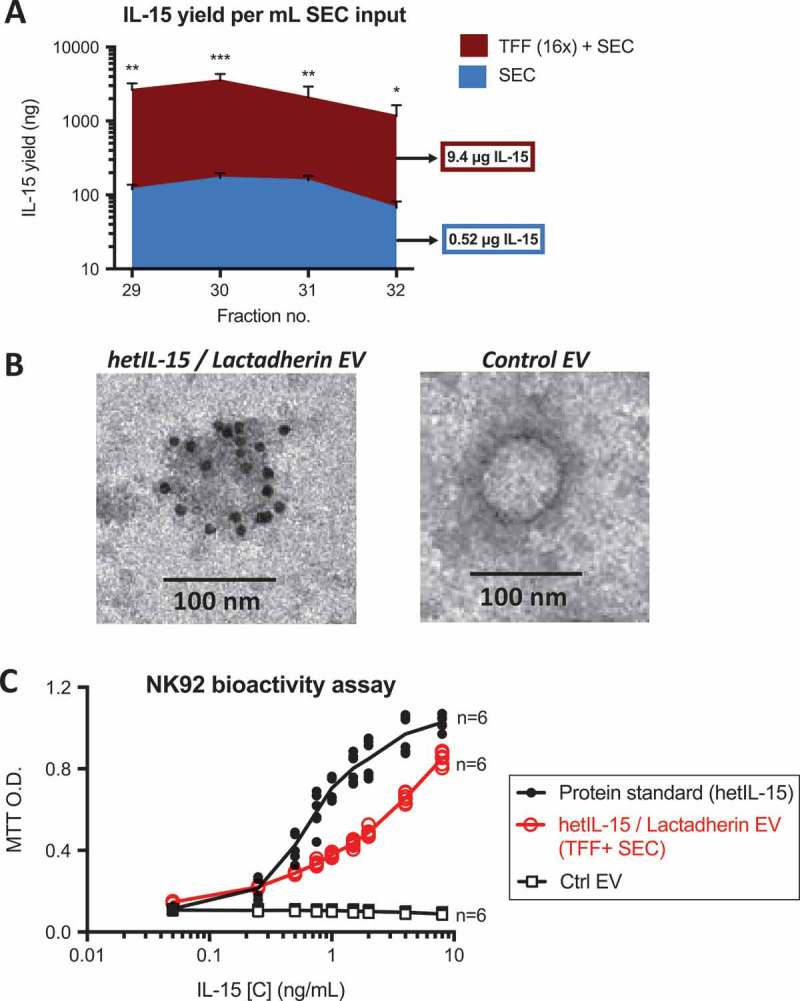
**TFF+SEC purifies multivalent, bioactive hetIL-15/lactadherin EV**. EV from HEK293 cells expressing hetIL-15/lactadherin were purified from bioreactor-conditioned medium by SEC using a large 60 cm column (3 independent runs). Where indicated, samples were concentrated by 16-fold using TFF prior to SEC purification, as shown in Figure 5. (a) Chromatography fractions were analyzed for IL-15 content by ELISA; shown here are F29-32, the EV fractions most enriched in both IL-15 and the EV-associated protein CD63 (see Figure 6(e) for relevant data). TFF+SEC proportionally increased the yield of IL-15 in these fractions, as plotted in Panel A. Total IL-15 yield of each preparation method is listed in respectively colored boxes (mean of 3 runs). Statistical analysis was by two-way ANOVA. ***, **, and * indicate *p *< 0.001, 0.01 and 0.05, respectively. (b) EV-association of cytokine was confirmed by immuno-TEM of EV purified by TFF+SEC (using anti-IL-15 antibody). Black dots in association with hetIL-15/lactadherin EV surface membrane (left panel) denote presence of secondary antibody conjugated to 10 nm gold particles. EV purified by SEC from conditioned media of HEK293 cells not expressing the cytokine were negative for immuno-TEM staining (right panel). (c) Bioactivity of purified hetIL-15/lactadherin EV was demonstrated *in vitro* by MTT assay of the NK92 cell line, which proliferate in a concentration-dependent manner when cultured in the presence of increasing amounts of hetIL-15 (purified protein or EV-associated).

Having confirmed that TFF + SEC allowed for highly efficient purification of EV from large volumes of bioreactor-conditioned medium, we wanted to assess the effect of this procedure on the quantity and bioactivity of EV-associated hetIL-15/lactadherin. We assayed EV-rich fractions for IL-15 content, and found that 10 μg of EV-associated IL-15 was eluted per mL of chromatography column input when using TFF-concentrated (16×) conditioned medium, which was about 17.5 times more than eluted per mL input of unconcentrated conditioned medium (Figure 7(a)). Given the high levels of hetIL-15 cytokine in the EV preparations, we hypothesized that several molecules of hetIL-15 are present on each individual vesicle. Indeed, by immune-TEM for human IL-15, we confirmed the presence of numerous hetIL-15/lactadherin complexes on the surface of EV (Figure 7(b)). These data suggested that the TFF process did not lead to significant loss of the interaction between hetIL-15/lactadherin and the EV surface. To ensure that bioactivity of EV-associated hetIL-15 was retained, we performed an *in vitro* MTT assay that assessed the proliferation of the human NK92 cell line in response to increasing concentrations of IL-15. EV-associated hetIL-15 led to a dose-dependent increase in NK92 cell proliferation, albeit to a slightly lesser extent than the assay standard (purified, clinical-grade hetIL-15 protein; Figure 7(c)). This could be due in part to the multivalency of cytokine-bearing EV, which may not allow every hetIL-15/lactadherin molecule on EV to ligate its receptor on responding cells due to steric hindrance. Nonetheless, our results show that hetIL-15/lactadherin EV purified by TFF + SEC are bioactive.

## Discussion

Methods of efficient, scalable, cGMP-compatible production and purification of EV are a prerequisite for clinical applications. In this study, we demonstrated that a combination of two cGMP-compatible technologies, namely TFF and preparative-scale SEC, produces EV of high purity and biological function.

As source material for our experiments, we used conditioned medium from HEK293 cells expressing a novel human construct of EV-associated hetIL-15/lactadherin. While fusion with the C1C2 domains of mouse lactadherin was previously used to increase the loading of EV with therapeutic proteins [18], we chose to test the ability of the homologous human sequence to achieve the same effect. In our study, we found that the chimeric human hetIL-15/lactadherin construct was significantly enriched on the surface of EV. Thus, the region of human lactadherin we identified as homologous to the C1C2 domain of mouse protein can be used to load the surface of EV with target proteins, which may be beneficial in reducing immunogenicity of therapeutic EV [36].

In addition to obtaining engineered EV from the cell line producing the lactadherin-fusion protein (as in our study), purified EV-free recombinant lactadherin-fusion proteins could be added to EV from primary cells, thus loading them with a therapeutic or targeting protein, as previously described [37]. This could comprise an efficient method for enhancing the activity of EV obtained from primary cells, such as dendritic or mesenchymal stem cells.

Regarding the production of EV, we previously reported the use of a lab-scale, hollow-fibre bioreactor to efficiently grow large numbers of cells in serum-free medium [15]. This method can be used to produce ~40-times more EV per mL medium than conventional cell culture, and in addition these preparations lack animal proteins present in serum-supplemented media. This platform was used in the current study, and allowed the production of several litres of serum-free, EV-rich medium to be generated. Besides being used to obtain EV from HEK293 cells, as in our study, hollow-fibre bioreactors have been used to differentiate bone marrow-derived mesenchymal stem cells, and to subsequently harvest EV [38]. Thus, hollow-fibre bioreactors comprise a useful source to obtain EV from both recombinant cell lines and primary cells with characteristics that are amenable to downstream purification. For applications that require larger-scale preparations (i.e. thousands of litres of conditioned media), the use of industrial-sized bioreactors in a cGMP-setting will likely be necessary.

Both in the case of hollow-fibre bioreactors and industrial-grade bioreactors, the volumes of conditioned culture medium that must be processed to obtain large EV preparations pose a challenge for downstream purification. Thus, in our scale-up experiments, a simple pump-driven TFF apparatus was used to rapidly remove non-EV components from bioreactor-conditioned media by isovolumetric filtration, and subsequently to significantly concentrate these media.

The ability to utilize large pore sizes without significant losses of EV is an important advantage of TFF. We took advantage of the large size of EV compared to other cell-secreted components by using a TFF module with 50 nm pores. Processing of conditioned medium with this filter decreased the concentration of non-EV components, including large ferritin complexes. Other methods, including U/C and SEC alone achieved far less efficient removal of these components, thus resulting in EV preparations of lower purity. In addition, removal of large amounts of non-EV macromolecules may be beneficial in allowing for increased concentration, by avoiding EV aggregation with protein complexes, and for decreased viscosity.

For some applications, additional processing of conditioned medium after TFF may not be necessary, given the removal of significant amounts of non-EV material. However, we found that subsequent SEC was able to further separate out non-EV macromolecular complexes (ferritin) in EV preparations, and to possibly discriminate between EV subsets (in our case eluting an early CD63-low fraction of EV). Thus, a final step of SEC processing may be important in applications requiring ultra-high purity preparations, such as in clinical development.

In our pilot and scale-up experiments with SEC, we used commercially available, pre-packed columns connected to a laboratory HPLC system to control and monitor EV elution. Our pilot experiments showed that the SEC purification alone was very efficient, yielding an equal number of EV as did U/C, which non-specifically pellets EV and large macromolecular complexes. For larger applications, industrial-grade SEC columns packed on-site could likely perform in a similar fashion as a final purification step. Moreover, following optimization of column parameters such as packing material, dimensions and flow rate, tandem injections of samples may be accomplished through parallel or interlaced column assemblies to further boost processing efficiency.

We also showed that TFF+SEC enables scalability of EV production, effectively eliminating the primary limitation of chromatography based purification methods. We began with purification of EV from 0.5 mL of bioreactor-conditioned medium using the 30 cm pilot-scale SEC column. By batched concentration of 220 mL of medium to ~14 mL using TFF (in approx. 1 h), and using a larger 120 mL SEC column, we were able to process the equivalent of 80 mL of bioreactor harvests in each 2-h chromatography run, yielding highly purified EV preparations containing 7.7 × 10^12^ vesicles per mL of input in the SEC column. While our midi-sized filters were rated at processing between 100 mL to 3 L volumes, maxi-sized filtration modules capable of handling more than 1000 L are commercially available. It is likely that processing of larger volumes will further increase the concentration capacity of this method, given the more favourable relationship between dead-space and starting conditioned medium volume. Notably, we did not detect significant losses of EV resulting from the TFF process, as 16-fold concentration of medium led to an estimated 30-fold increase in EV purified by subsequent SEC. The apparent increased recovery of EV following TFF may be a result of multiple factors, such as decreased losses within the SEC column and improved detection by NTA due to a higher vesicle concentration. Measurement of EV-associated cytokine also showed a significant increase in yield (18-fold versus unconcentrated medium), thus confirming the absence of significant EV losses by this procedure.

## Conclusion

Using the scalable production and purification workflow described in this study, we obtained large amounts of highly purified multivalent EV incorporating bioactive, fully human hetIL-15/lactadherin complexes. Future studies will test these EV as delivery vehicles of immunotherapy in preclinical models. The described methods are based on well-established technologies, which can be readily applied to generate purified preparations of engineered EV for research and development. Cost for the consumables required (TFF-devices, chromatography columns) is proportional to the volume of the starting material, making it an appealing workflow for various application scales. Confirmation of the utility of the method proposed in this study in industrial-scale production of EV remains to be demonstrated in an appropriate setting. Given the established use of TFF and SEC in industrial bioproduction, and their compatibility with cGMP settings, the proposed methodology comprises a promising candidate for production and purification of EV for clinical use.

## Supplementary Material

Supplemental_data.zip
