# Supplementary material for: Scalable, cGMP-compatible purification of extracellular vesicles carrying bioactive human heterodimeric IL-15/lactadherin complexes
Source: J Extracell Vesicles. 2018 Feb 28;7(1):1442088. doi: 10.1080/20013078.2018.1442088 (PMC5844027; doi:10.1080/20013078.2018.1442088)
Supplement: Supplemental_data.zip [file ZJEV_A_1442088_SM6568.zip › Supplemental data/Supplementary Figures-R2.docx]

**Supplementary Figures**

**Figure S1** Amino acid alignment of mouse and human lactadherin.

**Figure S2** hetIL-15 / Lactadherin fusion protein.

**Figure S3** Pump-driven TFF apparatus.

**Figure S4** EV elute in early fractions of SEC runs.

**Figure S5** Size distribution of EV is similar following SEC or UC purification.

**Figure S6** Comparative proteomics of SEC and U/C purified EV.

**Figure S7** First UV-absorbance peak of SEC corresponds to eluted EV.

**Figure S8** Individual measurements of particle and IL-15 yield in 60-cm column SEC fractions.

**Figure S9** EV-rich fractions from TFF+SEC lack cellular protein contamination, and express different amounts of CD63.

**Figure S10** Complete Western blots and TEM images from Figures 6, 7, and S9.

Mouse MQVSRVLAALCGMLLCASGLFAASGDFCDSSLCLNGGTCLTGQDNDIYCLCPEGFTGLVC 60

Human .PRP.L......A....PS.LV.--------------------------------L---- 24

Mouse NETERGPCSPNPCYNDAKCLVTLDTQRGDIFTEYICQCPVGYSGIHCETETNYYNLDGEY 120

Human -DI----..K...H.GGL.EEISQEV...V.PS.T.T.LK..A.N....K---------- 69

Mouse MFTTAVPNTAVPTPAPTPDLSNNLASR**CSTQLGMEGGAIADSQISASSVYMGFMGLQRWG** 180

Human ---------------------------.VEP....N.N..N...A....RVT.L...H.V 102

Mouse **PELARLYRTGIVNAWTASNYDSKPWIQVNLLRKMRVSGVMTQGASRAGRAEYLKTFKVAY** 240

Human ......N.A.M.....P.SN.DN.........R.W.T..V......LASH....A..... 162

Mouse **SLDGRKFEFIQDESGGDKEFLGNLDNNSLKVNMFNPTLEAQYIKLYPVSCHRGCTLRFEL** 300

Human ..N.HE.D..H.VNKKH...V..WNK.AVH..L.ETPV....VR...T...TA....... 222

Mouse **LGCELHGCSEPLGLKNNTIPDSQMSASSSYKTWNLRAFGWYPHLGRLDNQGKINAWTAQS** 360

Human .....N..AN.......S...K.IT........G.HL.S.N.SYA...K..NF...V.G. 282

Mouse **NSAKEWLQVDLGTQRQVTGIITQGARDFGHIQYVASYKVAHSDDGVQWTVYEE--QGSSK** 418

Human YGNDQ.......SSKE..........N..SV.F.......Y.N.SAN..E.QDPRT.... 342

Mouse **VFQGNLDNNSHKKNIFEKPFMARYVRVLPVSWHNRITLRLELLGC** 463

Human I.P..W..H.....L..T.IL.....I...A.....A........ 387

**Figure S1. Amino acid alignment of mouse and human lactadherin.**

Mouse (P21956) and human (Q08431) lactadherin amino acid sequences (also known as Milk fat globule-EGF factor 8; MFGE8) were obtained from [www.uniprot.org](http://www.uniprot.org) . Alignment revealed homologous regions, especially spanning the mouse C1C2 domains (indicated by **bold blue lettering**), which have been shown to bind to the surface of extracellular vesicles.

**B**

**A**

**MAPRRARGCRTLGLPALLLLLLLRPPATRGITCPPPMSVEHADIWVKSYSLYSRERYICNSGFKRKAGTSSLTECVLNKATNVAHWTTPSLKCIRDPALVHQRPAPPSTVTTAGVTPQPESLSPSGKEPAASSPSSNNTAATTAAIVPGSQLMPSKSPSTGTTEISSHESSHGTPSQTTAKNWELTASASHQPPGVYPQGPSYTCTCLKGYAGNHCETKCVEPLGMENGNIANSQIAASSVRVTFLGLQHWVPELARLNRAGMVNAWTPSSNDDNPWIQVNLLRRMWVTGVVTQGASRLASHEYLKAFKVAYSLNGHEFDFIHDVNKKHKEFVGNWNKNAVHVNLFETPVEAQYVRLYPTSCHTACTLRFELLGCELNGCANPLGLKNNSIPDKQITASSSYKTWGLHLFSWNPSYARLDKQGNFNAWVAGSYGNDQWLQVDLGSSKEVTGIITQGARNFGSVQFVASYKVAYSNDSANWTEYQDPRTGSSKIFPGNWDNHSHKKNLFETPILARYVRILPVAWHNRIALRLELLGC**

**Figure S2. hetIL-15 / Lactadherin fusion protein.**

(A) hetIL-15 / Lactadherin was expressed from a dual-promoter plasmid, encoding mature human IL-15 and the IL-15Rα ectodomain fused to the lactadherin C1C2 domains. (B) Amino acid sequence of IL-15Rα / Lactadherin fusion protein. IL-15Rα is indicated by **blue letters**, and lactadherin by **green letters**. IL-15Rα signal peptide is underlined.

**A**

**B**

**Figure S3. Pump-driven TFF apparatus.**

(A) TFF apparatus used for concentration of bioreactor media consisted of a commercial midi-scale TFF module connected to a sample reservoir by silicone tubing. Dialysate was connected to sample reservoir to allow for isovolumetric filtration. An analog pressure gauge was connected to ensure pressure developed by peristaltic pumping was maintained within operating parameters of the TFF module. Filtrate was collected as waste in a large flask. (B) Bioreactor medium in sample reservoir is shown before and after isovolumetric filtration with 4-volumes PBS.

**Chromatography fraction characterization**

**A**

**B**

**Figure S4. EV elute in early fractions of SEC runs.**

(A) Bioreactor conditioned medium was processed by SEC through a 24-cm preparative-grade Superdex column. UV-absorbance at 260 and 280 nm (A260 and A280, respectively) were monitored for the duration of the runs, measured in milli-absorbance units (mAU). (B) Fractions 8-15 (corresponding to 8-15 min of runtime) were analyzed by NTA to determine particle (EV) concentration. Fraction 9 had the majority of EV, coinciding with the first UV-absorbance peak. Ferritin is a 24-unit protein complex (approx. 8 nm in diameter) that was identified as a major protein contaminant of EV preparations purified by U/C (see **Figure S6**). The ability of SEC to resolve between EV and ferritin was assessed by ELISA. The majority was found to elute after EV (Fractions 10-13), allowing for efficient separation and augmented purification of EV.

**EV size distribution (additional independent preparation pairs)**

**Figure S5. Size distribution of EV is similar following SEC or UC purification.**

Size distribution was measured by NTA. Shown are comparisons of paired conditioned media, purified by U/C or SEC, in addition to representative example in **Figure 2B**.

**Comparative proteomics of SEC vs. U/C purified EV**


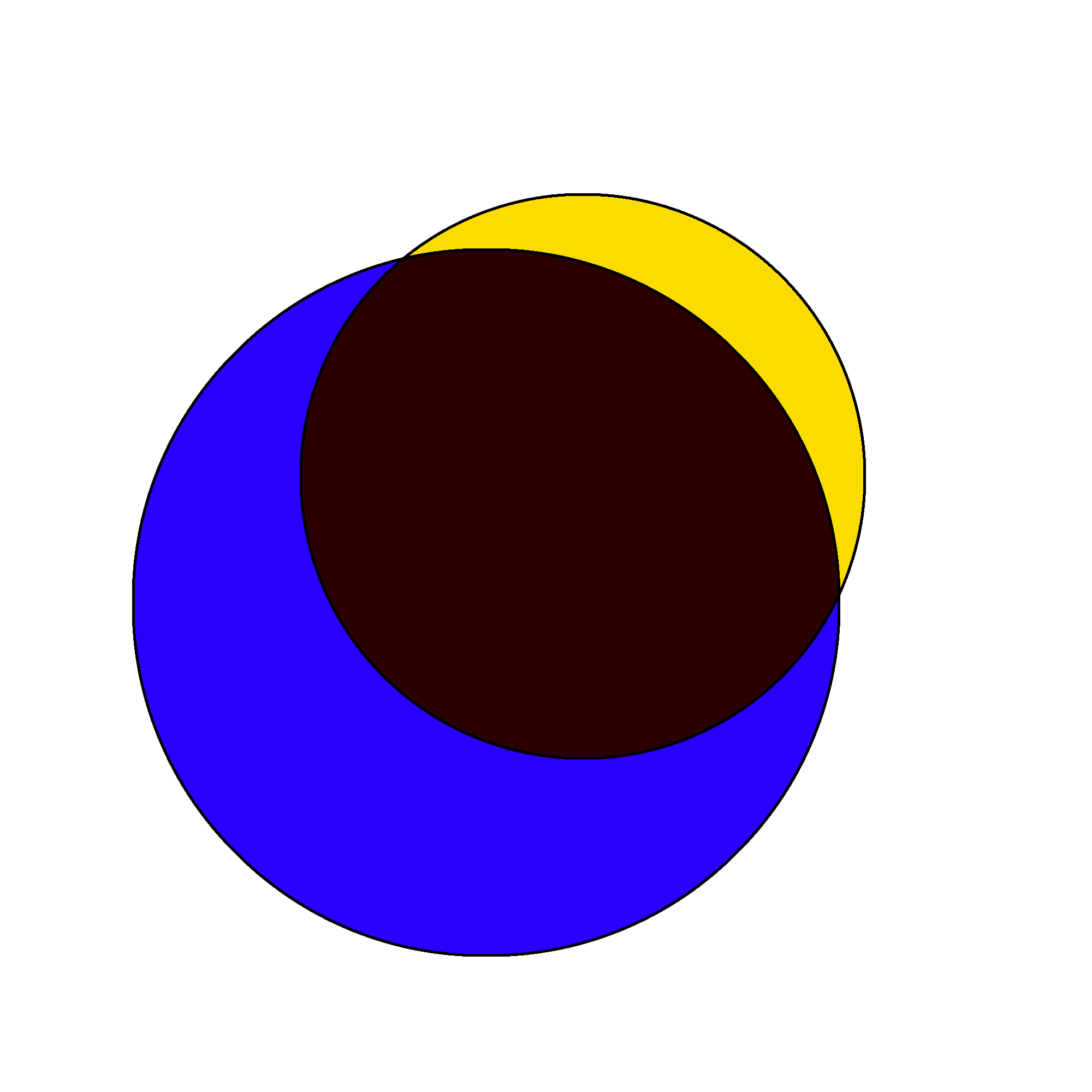


**SEC**

**U/C**

***574***

***668***

***135***

**B**

**A**

**Figure S6. Comparative proteomics of SEC and U/C purified EV.**

EV purified by either size-exclusion chromatography (SEC) or ultracentrifugation (U/C) were analyzed by high resolution mass spectrometry. Detected peptides were mapped to the human proteome. (A) Most of the proteins detected in SEC preparations were common to both purification methods, while U/C had a nearly equal number of unique hits. (B) Abundance of the majority of common proteins was similar between the two preparation methods, as shown in the correlation plot of peak area data for each mapped protein. The dotted line represents perfect correlation. Our analysis showed that the ferritin heavy and light chains (highlighted in magenta) were decreased by the greatest amount with SEC purification.

*On next pages:*

(C) Unique and shared mapped protein hits were used for a gene-set enrichment analysis (GSEA) using the PANTHER overrepresentation test, based on the GO cellular component ontology database (PMID: 27899595). In the following tables, we show the top 10 most significant cellular component ontologies (by *p*-value) enriched in each dataset. We found the enrichment of ontologies related to EV and exosomes to be highly significant among the both shared and unique protein hits. This was more so in the case of shared proteins. However, in the case of U/C unique proteins, enrichment of other cellular component ontologies was more significant.

Complete proteomics and analysis data can be found in **Supplementary Data 1**.

--------- Continued on next page ---------

**C**

**First UV-absorbance peak of SEC corresponds to eluted EV**

**Figure S7. First UV-absorbance peak of SEC corresponds to eluted EV.**

A minor tubing-length change in our SEC-apparatus resulted in a 1-minute shift of eluted fractions in the pilot-scale TFF experiments. We re-confirmed that the first UV-absorbance peak (F8) corresponds to the main EV peak, by analyzing Fractions 7-11 in one sample. Bars represent mean ± SEM of 5 technical replicates.

**A. Particle yield per mL SEC input**

| ***Fraction no.*** | **SEC alone** | | | **TFF+SEC** | | |
| --- | --- | --- | --- | --- | --- | --- |
| 27 | 3.84E+09 | 8.99E+09 | 4.00E+09 | 6.85E+11 | 1.12E+11 | 1.66E+11 |
| 28 | 3.74E+10 | 2.20E+10 | 3.90E+10 | 5.91E+11 | 2.17E+12 | 1.61E+12 |
| 29 | 4.65E+10 | 5.76E+10 | 4.84E+10 | 2.45E+12 | 3.74E+12 | 2.37E+12 |
| 30 | 5.76E+10 | 8.19E+10 | 6.00E+10 | 1.12E+12 | 2.33E+12 | 1.25E+12 |
| 31 | 6.22E+10 | 5.22E+10 | 6.48E+10 | 9.98E+11 | 1.06E+12 | 4.21E+11 |
| 32 | 1.82E+10 | 2.07E+10 | 1.89E+10 | 9.01E+11 | 3.35E+11 | 2.01E+11 |
| 33 | 4.74E+09 | 6.69E+09 | 4.93E+09 | 2.62E+11 | 1.36E+11 | 6.90E+10 |
| 34 | 1.87E+09 | n.a. | n.a. | 5.31E+10 | 4.26E+10 | 1.25E+10 |

**B. IL-15 yield (ng) per mL SEC input**

| ***Fraction no.*** | **SEC alone** | | | **TFF+SEC** | | |
| --- | --- | --- | --- | --- | --- | --- |
| 29 | 136.6 | 139.2 | 83.4 | 3541.2 | 1526.8 | 2840.3 |
| 30 | 166.4 | 216.8 | 130.1 | 5077.1 | 2478.1 | 3063.6 |
| 31 | 168.5 | 191.0 | 114.4 | 3701.2 | 1681.7 | 832.9 |
| 32 | 89.9 | 70.4 | 42.3 | 2057.3 | 966.2 | 491.0 |

**Figure S8.** **Individual measurements of particle and IL-15 yield in 60-cm column SEC fractions.**

(A) Summary of individual measurements (by NTA) of EV particle concentration in chromatography fractions eluted in SEC alone of TFF+SEC experiments. These data were plotted in **Figure 6A**. Each measurement above represents the mean of 5 technical replicates analyzed by NTA. “n.a.” indicates not available. (B) Summary of individual measurements (by ELISA) of IL-15 eluted in CD63^+^ EV chromatography fractions of the same experiments. These data were plotted in **Figure 7A**.


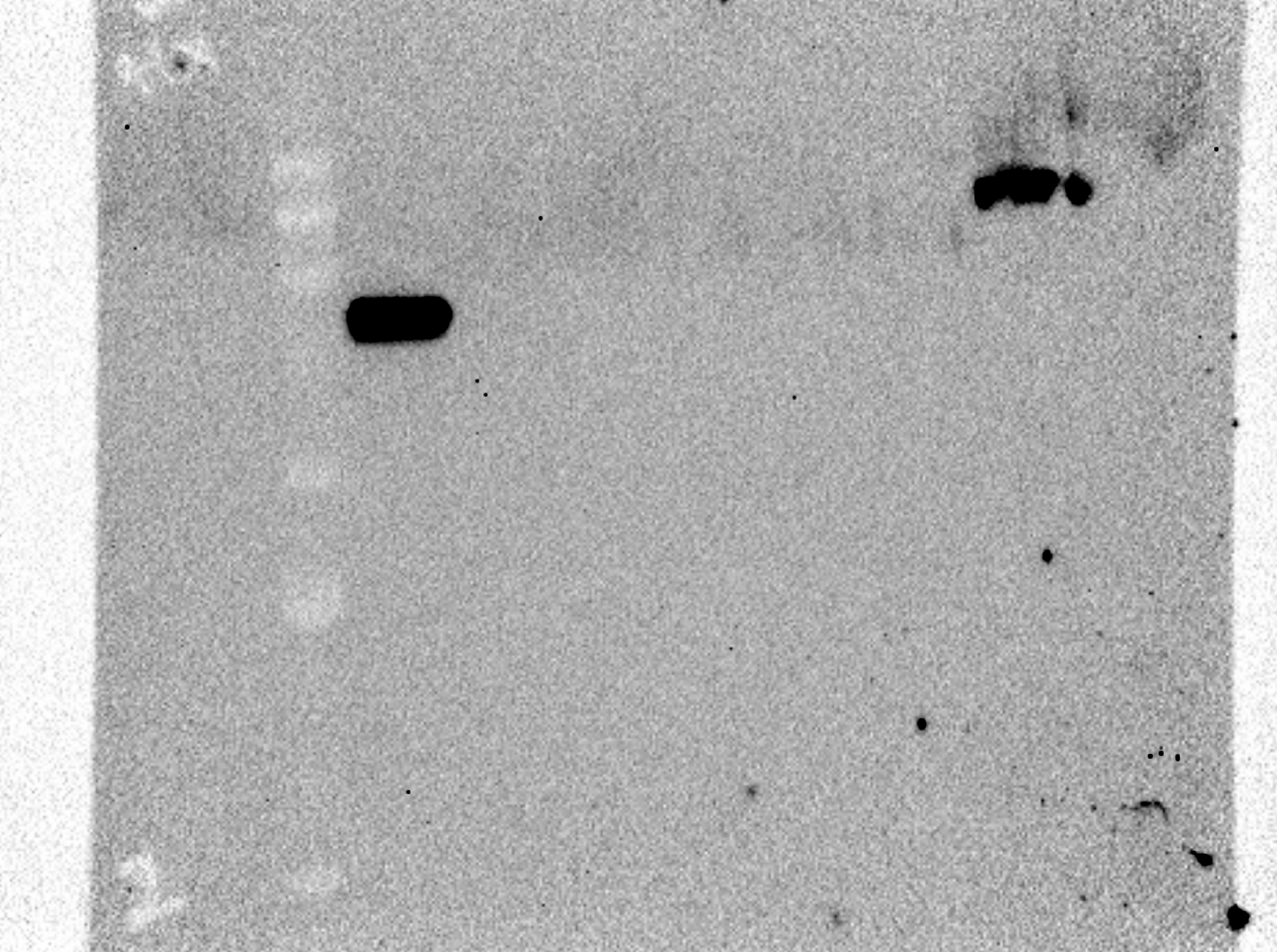

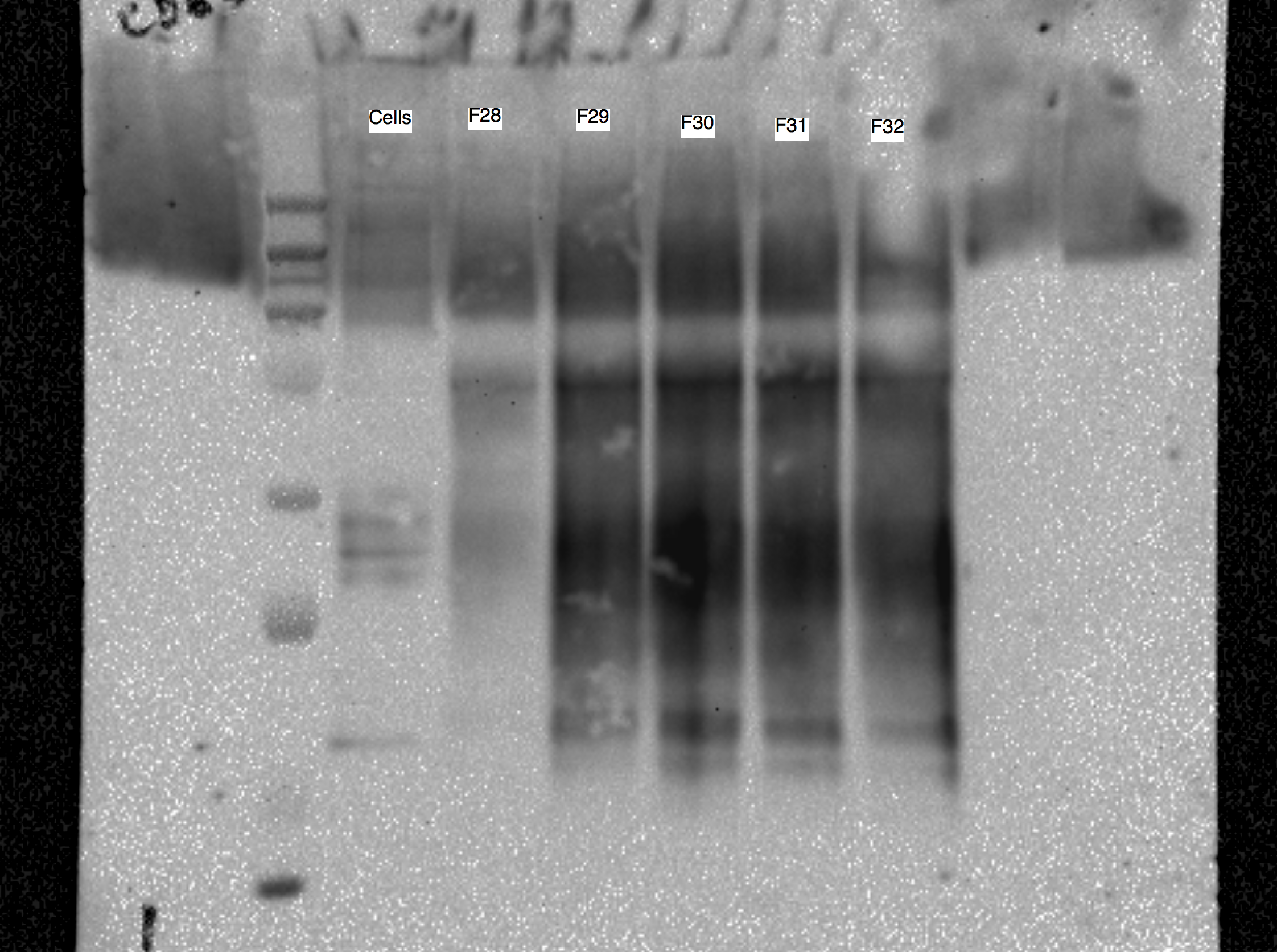


**Cell**

**F28**

**F29**

**F30**

**F31**

**F32**

75 kDa --

50 kDa --

37 kDa --

20 kDa --

75 kDa --

100 kDa --

*CD63*

*Calnexin*

**Figure S9: EV-rich fractions from TFF+SEC lack cellular protein contamination, and express different amounts of CD63.**

Bioreactor-conditioned medium were concentrated 16-fold by TFF and then EV were purified by SEC using a large 60 cm column. Equal protein amounts of EV-rich chromatography fractions (see **Figure 6**) were analyzed by Western blot to assess EV composition normalized to total protein. Calnexin (cell-associated protein) could not be detected in EV-rich fractions with high levels of CD63 (EV-associated protein). The first EV fraction (F28) appeared to have a lower abundance of CD63, suggesting a different subtype of vesicle compared to subsequent fractions.

**Uncropped gels and TEM images**


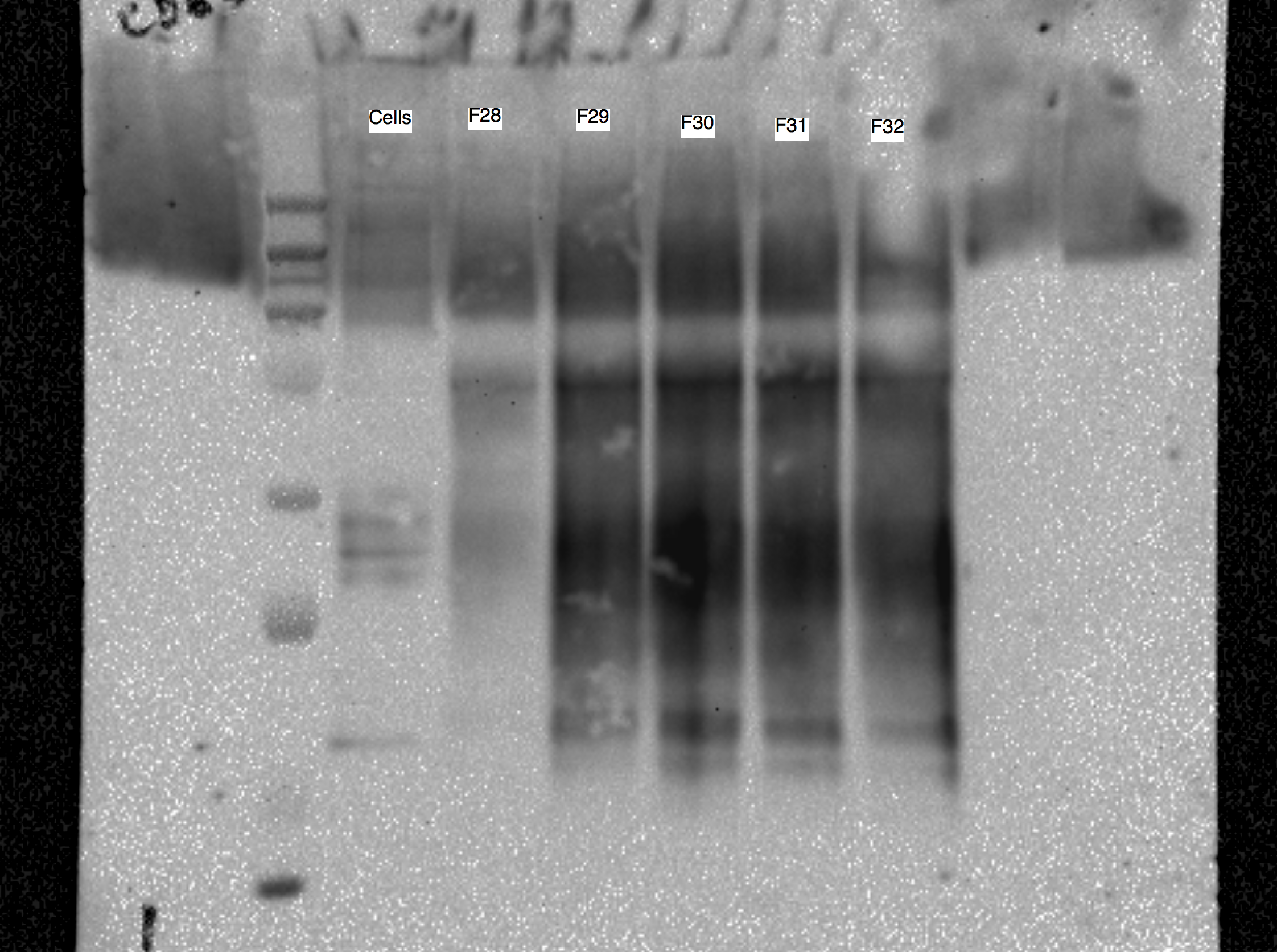


**Cell**

**F28**

**F29**

**F30**

**F31**

**F32**

*CD63*

**Marker**

*Calnexin*

**Cell**

**F28**

**F29**

**F30**

**F31**

**F32**

**Marker**

**A**

**B**


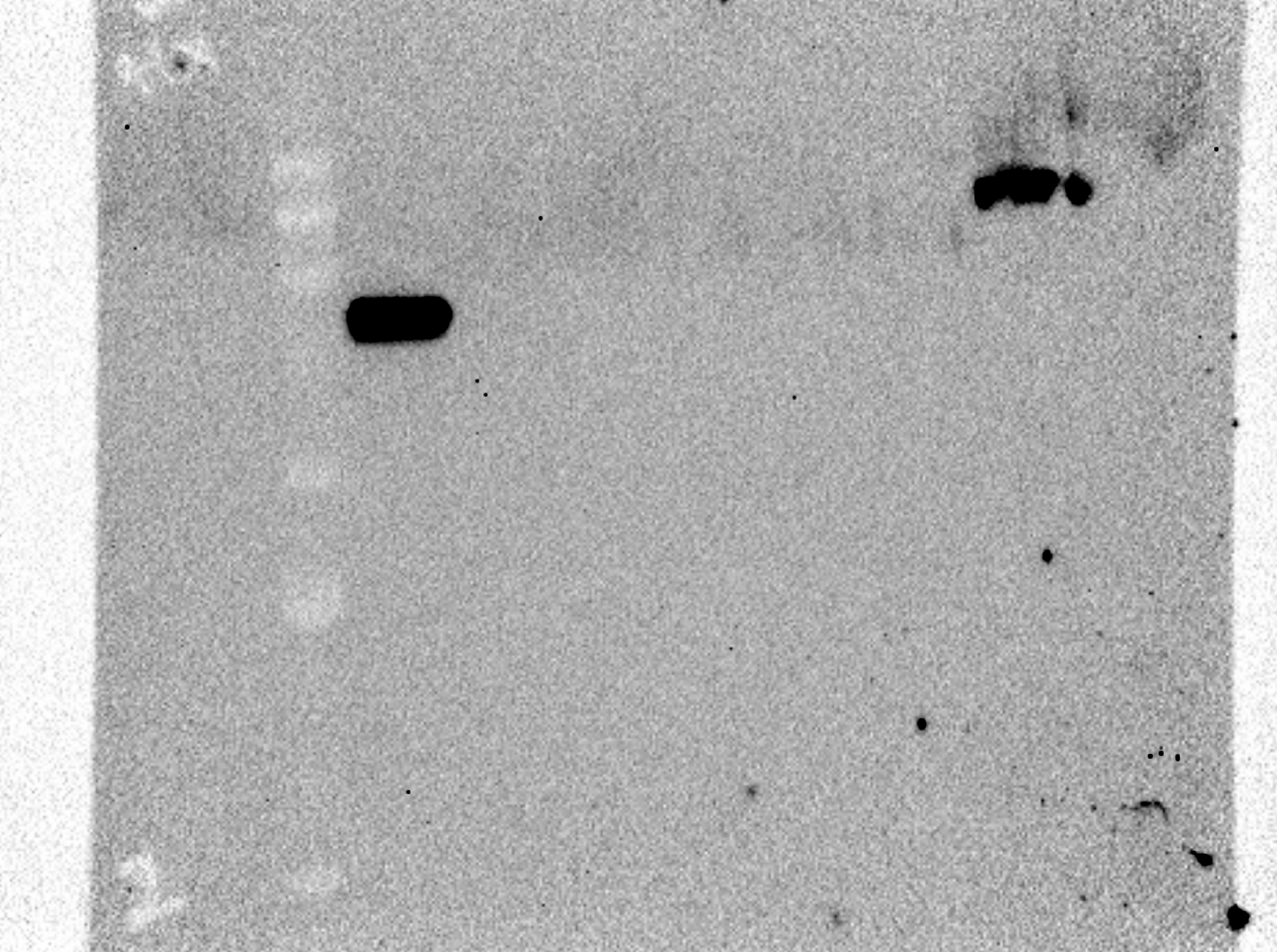


**C**

**F27**

**F28**

**F29**

**F30**

**F31**

**F32**

**F33**

**F34**

**F35**


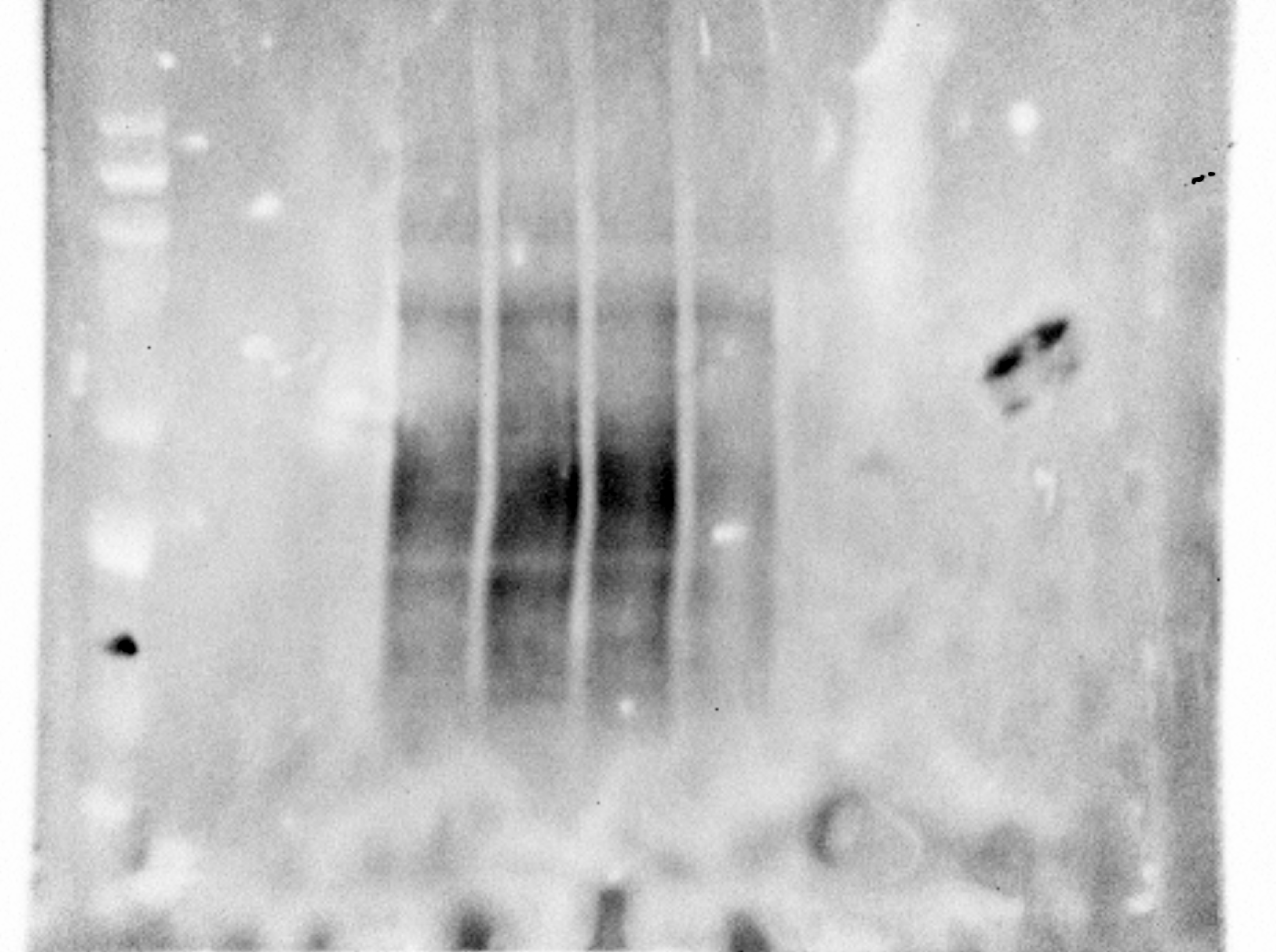


*CD63*

**hetIL-15 / Lactadherin EV**

**Control HEK293 EV**


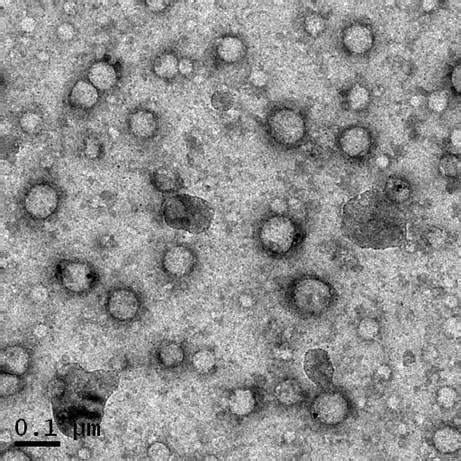

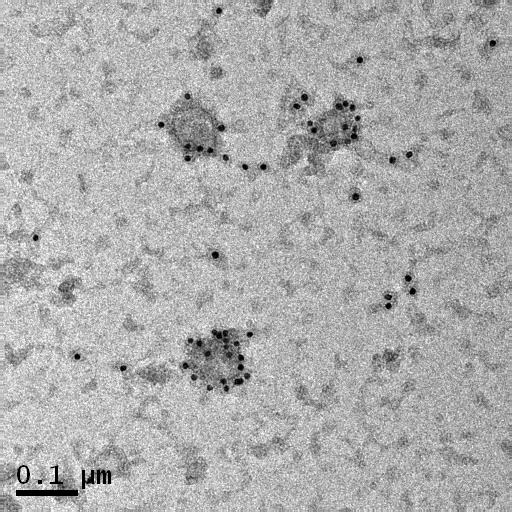


**D**

**E**

**Figure S10.** **Complete Western blots and TEM images from Figures 6, 7, and S9.**

(A) Western blot of TFF+SEC EV-rich fractions probed with anti-CD63 (blot from Figure S9; equal protein amount loading). (B) Western blot of TFF+SEC EV-rich fractions probed with anti-Calnexin (blot from Figure S9; equal protein amount loading). Blot was purposefully overexposed to maximize detection of any low-level calnexin presence in SEC fractions. (C) Western blot of TFF+SEC EV-rich fractions probed with anti-CD63 (blot from Figure 6E; equal fraction volume loading). (D) Immuno-TEM of hetIL-15 / Lactadherin EV (TFF+SEC), probed with anti-IL-15 (from Figure 7B). Black dots correspond to gold particle-conjugated secondary antibody. Most detectable hetIL-15 was associated with EV. Few detected EV-independent hetIL-15 molecules may correspond to unbound cytokine and/or fragments of EV generated as part of the TEM sample fixation and staining procedure. (E) Background immuno-staining of anti-IL-15 TEM was negligible, as shown by absence of gold nanoparticle signal in control EV from HEK293 cells lacking hetIL-15 expression (from Figure 7B).
